# Evaluation of Postoperative Pain in Response to Polydimethylsiloxane and Calcium Silicate-Based Endodontic Sealers Using the Visual Analog Scale (VAS)

**DOI:** 10.7759/cureus.48331

**Published:** 2023-11-05

**Authors:** Shweta Sedani, Utkarsh Umre, Simran Kriplani, Pradnya Nikhade

**Affiliations:** 1 Conservative Dentistry and Endodontics, Sharad Pawar Dental College and Hospital, Datta Meghe Institute of Higher Education and Research, Wardha, IND

**Keywords:** root canal therapy, post operative pain management, visual analog scale (vas), bioceramic, root canal sealer apical sealing ability

## Abstract

Background: The main purpose of obturation is to achieve a complete three-dimensional sealing of the pulp space to create a tight seal and prevent bacterial movement and its toxins to the periapical tissues. Different approaches and sealants have been developed due to the root canal system's intricacy for ensuring tight adherence. The root canal sealants need to establish a bond between the material and root dentine in order to prevent leakage. Even though the biocompatibility and sealing abilities of the materials are prioritized in modern endodontics, some sealers incorporate therapeutic or antibacterial drugs like corticosteroids or calcium hydroxide. An endodontic sealer's cytotoxicity and antibacterial capabilities must be perfectly balanced. Due to the limited evidence in endodontic literature regarding the relationship between postoperative pain and the sealers that are used in this study, we conducted the research to explore the same.

Aim: This study aimed to evaluate and compare pain in responses to polydimethylsiloxane and calcium silicate-based endodontic sealers (CS-BG).

Methodology: The participants were divided into two groups, Nanoseal (Nanoseal-S™; Prevest, DenPro, USA) and Bioceramic (CeraSeal™; Mera Biomed Co., Cheongju, Korea) with 20 patients in each group. After caries excavation and access opening, the biomechanical preparations were performed in each tooth. To avoid over-instrumentation, the working length was verified after each instrument use. The standard irrigation protocol was performed. Sealers were mixed according to the manufacturer's instructions followed by obturation. The patients were asked to take medicines only if they had pain. The pain was evaluated using the visual analog scale (VAS) postoperatively at 24 hours, 48 hours, and a week after the root canal obturation.

Results: Pain perception using Nanoseal-S™ and CeraSeal™ materials were compared at 24 hrs, 72 hrs, and seven days. Nanoseal showed statistically significant repletion at 24 hrs and seven days.

Conclusion: The decreased irritation character of CS-BG for the periapical tissues explains the lesser percentage of pain during and pain immediately after root canal obturation observed in this investigation, which proves CS-BG is highly biocompatible with periapical tissue and further reduces patient anxiety during root canal obturation.

## Introduction

Obturating the root canal space which has been prepared properly is one of the essential factors for a successful root canal procedure. Gutta-percha and sealer are helpful means for obturating the canal space [1]. Inadequate sealing of the root canal system is one of the most important reasons for endodontic failures. Obturation's main goal is to accomplish a complete hermetic sealing of the pulp space to create a tight seal and prevent bacterial movement and its toxins to the periapical tissues. Various approaches and sealants have been developed due to the root canal system's intricacy for ensuring tight adherence. The root canal sealants need to establish a bond between the material and root dentine in order to prevent leakage [2].

Although the biocompatibility and sealing abilities of the materials are prioritized in modern endodontics, some sealers incorporate therapeutic or antibacterial drugs like corticosteroids or calcium hydroxide. The cytotoxicity and antibacterial capabilities of an endodontic sealer should be considered [3].

Root canal sealants can potentially inhibit the repair of periodontal tissues through the root end and lateral canals as well as the periodontium [4]. Postoperative pain may be induced by the localized inflammation that root canal obturation materials induce. It has been related to a variety of treatment parameters, including root canal sealer selection, number of visits, measurement of working length (WL) with an apex locator, and instrumentation selection [5].

The composition of the sealer, in particular, has a significant impact on the severity of the inflammatory reaction and activates ions of sensory neurons. Also, sealers may cause postoperative pain after the completion of therapy. Sealers used in root canal treatment might activate trigeminal nociceptors; consequently, this activation and the immunologic response might trigger symptoms of pain and may flare up [6].

Bioceramics are biocompatible ceramic or metal oxide materials with improved sealing properties, and antibacterial, and antifungal activity used in dentistry. A root canal sealer incorporates nanosilver in chemical form, NanoSeal-S™ (Prevest, DenPro, USA) is a flowable polydimethyl siloxane. It has outstanding radioopacity and a great ability to seal. Chemically adding silver serves as a preservative. Due to the limited evidence in endodontic literature regarding the relationship between postoperative pain and the sealers used in this study, we conducted research to explore the same. So we aimed to evaluate and compare, using a VAS scale, the post-operative pain in responses to polydimethylsiloxane and calcium silicate-based endodontic sealers (CeraSeal™; Mera Biomed Co., Cheongju, Korea) [7].

## Materials and methods

This observational study was conducted in the Department of Conservative Dentistry and Endodontics, Sharad Pawar Dental College, Sawangi (Meghe), Wardha, India. The Institutional Ethics Committee of Datta Meghe Institute of Medical Sciences (DMIMS(DU)/IEC/2022/881) approved the study protocol. The patients visiting the Outpatient Department (OPD) participated in the study. All patients were informed thoroughly regarding the procedure and the materials used for the study. Written consent was obtained from them before the treatment.

Inclusion Criteria

The study included participants who were symptomatic and above 18 years old. These patients were referred for endodontic treatment. Also, the patients having more than one tooth indicated for Root Canal Therapy (RCT) were considered multiple samples. For them, however, the treatment was conducted at the interval of 14 days.

Exclusion Criteria

The study excluded the participants with teeth requiring re-treatment, root fracture, a badly mutilated tooth that cannot be isolated with a rubber dam, patients under 18 years of age, teeth responding positively to pulpal sensitivity test, and patients with systemic diseases.

Methodology

The participants were divided randomly into two groups of 20 patients each. A total of 40 patients having preoperative pain and indicated for root canal treatment, two groups of 20 subjects each, For Group I, Nanoseal (Nanoseal-S™) was used, whereas for Group II, Bioceramic (CeraSeal™) was used.

The entire procedure was performed under rubber dam isolation. Caries were excavated, and access opening was done using round bur and further, the access opening was refined using safe end bur. The pulp was extirpated with a suitable barbed broach. Then, the working length was established with the apex locater and confirmed using an intraoral periapical radiograph. Path finder files were used to create a glide path. Biomechanical preparation was performed using the ProTaper Gold system (PTG, DentsplyMaillefer, Ballaigues, Switzerland) and the X-Smart endodontic system (DentsplyMaillefer, Ballaigues, Switzerland). An X-Smart endodontic motor was used for the endodontic filing, to avoid over-instrumentation and the working length was verified after using each instrument. Two mL of 2% sodium hypochlorite (NaOCl) was flushed after each instrumentation. The master apical file that was used in the study ranged from F1 to F3 according to the dimensions of the apical foramen. Two mL 2% sodium hypochlorite (NaOCl) was ultrasonically activated for 30 seconds. The final rinse used was normal saline. Master cone fit was checked clinically and radiographically. The final irrigation was done using 2% chlorhexidine (CHX). Canals were dried using appropriate absorbent points till they were dry. Sealers that were used were mixed according to the instructions provided by the manufacturer. Then, the obturation was done. Alcohol was used to clean the pulp chamber to ensure the complete removal of the endodontic sealer. Each tooth was then post-endodontically restored with a composite restoration. The final radiograph was then obtained after the removal of the rubber dam. The medications were not prescribed to the patient, until and unless in the event of pain; they were instructed to take only paracetamol and diclofenac sodium. The intensity of evaluation of postoperative pain was registered postoperatively at 24 hours, 48 hours, and a week after the root canal obturation. The patients were provided with a Visual Analogue Scale (VAS) and instructed to record pain intensity based on their pain experience at that particular time. Scores from 1 to 10 were attributed. Any need for analgesics was recorded.

Statistical Analysis

The mean and standard deviation were used to express all parametric particulars. Multivariate analysis of variance in order to juxtapose the postoperative pain scores between groups and to identify changes over time, repeated measurements were used. StatPlus:mac software (Version v7; Analyst SoftInc, Walnut, CA) was used to statistically analyze the data.

## Results

Tables 1 and 2 depict the comparison of the pain perception in the patients treated endodontically with Nanoseal and Bioceramic material as a sealer. The statistically discernible difference in p-values was observed when both sealers were compared at intervals of 24 and 72 hours. Statistically significant repletion was shown in the Nanoseal and Bioceramic groups. In the Nanoseal group, the pain threshold was high after 24 hrs of obturation (2.25 ±1.74) followed by 72 hours (0.50±0.88). Least pain was observed in patients after seven days of obturation (0.25±0.55). The same results were shown by Bioceramic groups where the pain threshold was high after 24 hrs of obturation (0.25±0.55) followed by 72 hours of obturation (0.10±0.30).

**Table 1:** Comparison of Nanoseal and Bioceramic sealer by unpaired T-test Shows the comparison of the pain perception in the patient treated endodontically with Nanoseal and Bioceramic material as a sealer. A statistically discernible difference in p-value (less than 0.01) was observed when both sealers were compared at interval of 24 hours and 72 hours. For seven days, the data could not be computed because the standard deviations of both groups are 0.

Variables		N	Mean	Std. Deviation	Std. Error Mean	95% Confidence Interval		T value	p-value
						Lower	Upper		
24hrs	Nanoseal	20	2.25	1.74	0.38	1.17	2.82	0	4.89
	Bioceramic	20	0.25	0.55	0.12	1.15	2.84		
72 hrs	Nanoseal	20	0.25	0.55	0.12	0	0.49	0	2.03
	Bioceramic	20	0	0	0	0	0.5		

**Table 2:** Comparison of pain perception using both sealers by Post Hoc Tests Comparison of pain perception using Nanoseal and Bioceramic materials used during endodontic treatment at 24hrs, 72 hrs, and 7 days. Nanoseal showed a statistically significant p-value that is less than 0.01 and significant repletion at 24 hrs and seven days.

Variable			Standard Error	p-Value		
					95% Confidence Interval	
					Lower Bound	Upper Bound
Nanoseal s	24hrs	72 hrs	0.33	<0.001	1.33	2.66
		7 days	0.33	<0.001	1.58	2.91
	7 days	24hrs	0.33	<0.001	-2.66	-1.33
		7 days	0.33	0.45	-0.41	0.91
	72 hrs	24hrs	0.33	<0.001	-2.91	-1.58
		72 hrs	0.33	0.45	-0.91	0.41
Bioceramic	24hrs	72 hrs	0.1	0.01	0.04	0.45
		7 days	0.1	0.01	0.04	0.45
	72 hrs	24hrs	0.1	0.01	-0.45	-0.04
		7 days	0.1	1	-0.2	0.2
	7 days	24hrs	0.1	0.01	-0.45	-0.04
		72 hrs	0.1	1	-0.2	0.2

## Discussion

The occurrence or cause of the pain cannot be attributed to any certain factor, as root canal treatment (RCT) consists of tedious and multiple steps. RCT involves chemically and mechanically removing the structures infected by bacterial toxins and obturation of root canals. After clinical affirmation obturation of the prepared root canal space was completed in the same appointment, as the root canal was clean and had relatively less pathogenic load eventually as a result of the bio-mechanical preparation [8]. The occlusion of dentinal tubules is obtained using sealant by applying it to the master cone. Thus, the endodontic sealer and the technique of occluding the dentinal tubules of the root canal system may become the cause of pain. In the present study, for postoperative pain assessment, the visual analog scale (VAS) was used. It is an acceptable procedure for measuring postoperative pain in research related to dentistry. Otherwise, VAS is inculcated for recognizing the relative differences in acute and chronic pain. Also, it is important that in young and older patients, the scale used for assessing the pain makes it easier to use instrumentation further [9]. It was proven in earlier studies that the pain post-endodontic treatment is mostly specific to multirooted teeth and gender.

Gender differences have been emphasized by differences in the pain reaction to the stimulus. But, the current study showed gender and the type of tooth may not affect the occurrence of pain during and immediately after root canal obturation [10]. Most of the cases of “Pain” immediately after root canal obturation were due to perforation of the root canal apex, which indicates that a huge area of contact between the sealer and tissues presents periapically induced pain physiochemically. Otherwise, the effect of patient age has a substantial correlation with both the occurrence of pain during root canal obturation and the pain that follows promptly. However, since there are no reports of evidence demonstrating how aging affects pain perception, this conclusion is not clinically meaningful [11]. This study's pre-operative symptom list includes signs like percussion sensitivity, pain on biting, condition of periodontium, and swelling. The presence of pain during root canal obturation (PD) and pain immediately after root canal obturation (PI) was unaffected by the positive and negative symptoms as well as the diagnosis. According to the study results, the patients receiving single visit root canal therapy and experiencing the pain before the procedure are more likely to anticipate and report the pain after the procedure as well. Therefore, this study demonstrates that the incidence of pain during root canal obturation and discomfort just after root canal obturation is not substantially correlated with the improvement of the patient's complaints. The occurrence of pain during root canal obturation (PD) and pain immediately after root canal obturation (PI) was unaffected by the tooth's original or subsequent treatment.

Furthermore, there was a significant difference in statistics of the incidence of PD depending on the endodontic treatment method [12]. The specific cells that contributed the most to the Chi-square test result were discovered by residual analysis. The investigation revealed that during root canal obturation, infected root canal-treated teeth marginally increased "Discomfort." Further, it was also discovered that the root apex of these "Discomfort" teeth was open. A tooth's opened root apex makes it simple for a root canal sealer to apply mechanical pressure. However, endodontic therapy had no impact on the occurrence of pain immediately after the obturation. Treatment of root canals impacting PI was not conclusively demonstrated in the prior study. The various obturation materials and techniques, as well as treatment approaches, may be to blame for the prevalence of PI [10]. Techniques used during root canal obturation had no discernible impact on the prevalence of PD and PI. The physicochemical properties of CS-BG were linked to the reaction of pain during the obturation of the root canal and pain immediately post-obturation of the root canal [13]. In contrast to other sealers, such as resin-based, bioceramics-based, eugenol-based, and non-eugenol-based sealers, our investigation showed that the "Pain" (0.5%) level of PI responded by CS-BG was relatively lower. Additionally, it is said that sealers have an irritating impact that is gradually reduced with time [14].

Other cases of “Discomfort” and “Pain” levels immediately after root canal obturation had no symptoms within a week. In an earlier study, it was emphasized that eugenol has a caustic effect and that resin during the early curing phase depicts similar effects. Rest other states that, due to its rather unstable physical characteristics, the bioceramic-based sealer exhibits significant cytotoxicity in its freshly combined condition. The physicochemical stability and biocompatibility of CS-BG are thought to be responsible for the low proportion of pain experienced during root canal obturation and pain experienced right away after root canal obturation that was seen in this study. The beginning of postoperative pain, however, is said to be unaffected by these sealers in any other way. The findings of this study agree with those of the earlier ones. It would be useful to compare and contrast the occurrence of postoperative discomfort following root canal obturation with CS-BG and all the other sealers in the longer-term plan [15].

## Conclusions

The study participants reported only 1% and 0.5% of "Discomfort" and "Pain" in their CS-BG-obturated teeth, respectively. The decreased irritation character of CS-BG for the tissues, present periapically may explain the lesser percentage of pain during and pain immediately after root canal obturation as observed in this investigation. These clinical findings demonstrate that CS-BG is highly biocompatible with periapical tissue thus reducing patient anxiety during root canal obturation. A clinical trial would eventually offer a most relevant clinical study, which would cater to a relatively increased level of proof for evaluating the CS-BG when compared to the other sealers being used.
